# CRISPR/Cas9 can mediate high-efficiency off-target mutations in mice in vivo

**DOI:** 10.1038/s41419-018-1146-0

**Published:** 2018-10-27

**Authors:** Neeraj K Aryal, Amanda R Wasylishen, Guillermina Lozano

**Affiliations:** 1 Department of Genetics, The University of Texas MD Anderson Cancer Center, 1515 Holcombe Blvd., Houston, TX 77030 USA; 2 Genes and Development Program, The University of Texas MD Anderson Cancer Center UTHealth Graduate School of Biomedical Sciences, 6767 Bertner Avenue, Houston, TX 77030 USA

The CRISPR/Cas system has revolutionized the field of genome-editing as it is easier, faster, less expensive, and more efficient than traditional methods. In a very short time, its applications have become very diverse and widely used. Recently, this technology has been applied in human embryos to correct pathogenic mutations and has re-ignited the ethical debate of germ-line editing^1,2^. Besides the ethical considerations, the possibility of creating off-target mutations with unknown consequences is a concern.

There are many reports of high-frequency off-target mutations in human and mouse cell-lines^3,4^, but unwanted errors in mammalian embryo editing have been deemed as a rarity^5–9^. In a study with human embryo editing, one embryo was profiled for off-target mutations by whole-genome sequencing^2^. Even though they report no off-target mutations, the number is too small (*n* = 1) for statistical significance and the on-target locus has 1 mismatch with the sgRNA used. Previous studies with mouse embryos have reported rare and low-frequency mutations at sites with >1 mismatches^8,10^. Here we report high-frequency mutations at an off-target site with three mismatches using this gene editing technology in the mouse embryo.

We targeted *Dicer1* to introduce a mutation at a Serine residue by pronuclear injection of sgRNA (7.5 ng/µl), spCas9 mRNA (10 ng/µl), and a 123 base single-stranded donor oligo (20 ng/µl) into mouse embryos using previously described methodology^11^. The sgRNA has a quality score of 76 (out of 100) with 124 potential off-target sites according to the crispr.mit.edu site. In all 38 mice from 200 injected zygotes, we used the polymerase chain reaction to amplify and Sanger sequence a 1 Kbp region of the on-target and the top two candidate off-target sites that contain a proto-spacer adjacent motif (PAM) site. We observed the on-target efficiency of 40% (15/38 animals) with non-homologous end joining (NHEJ) being the predominant repair mechanism (Fig. 1a). Only 5% of the mice (2/38) had the intended mutation introduced by homologous recombination (both were mosaic based on sequencing and validated by genotyping of F1 progeny). Surprisingly, at the top predicted off-target site (intergenic) with 3 mismatches at positions 1, 4, and 8 (where 1 is farthest from the PAM site), we observed mutations in 29% (11/38) of mice (Fig. 1b). Off-target mutations at this site were observed in 3 mice (#1, #2, and #3) that did not have mutations at the on-target site. Additionally, bi-allelic off-target mutations were observed in 2 mice (#4 and #5). At the second candidate off-target site with 4 mismatches at positions 1, 2, 5, and 8 followed by a PAM, sequencing results did not reveal mutations in any of the 38 mice. We further evaluated 3 additional off-target sites in six animals (with on-target mutations) and identified no additional mutations. Since *Dicer1* is an essential gene, bi-allelic mutations may result in embryonic lethality. As a result, we may have missed additional on- and off-target mutations.

**Fig. 1:**
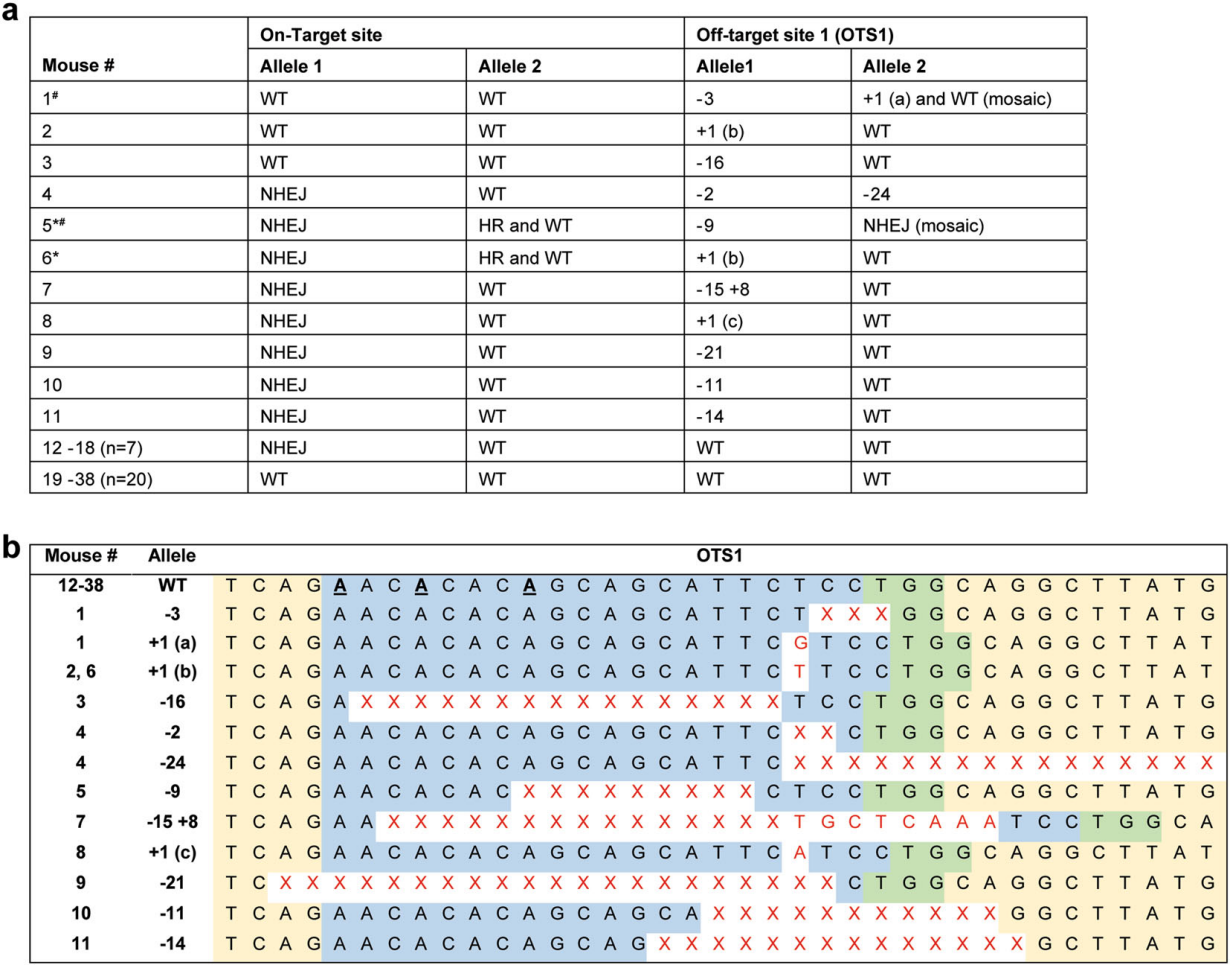
On- and off-target changes observed for all 38 mice born from CRISPR/Cas9 targeted embryos. **a** Allelic description for all mice at both on- and off-target sites. *, mosaic at on-target site; ^#^, mosaic at off-target site; WT, wild-type; NHEJ, non-homologous end-joining; HR, homologous recombination. **b** List of all mutations observed at an off-target site with three mismatches. Off-target site is in blue, mismatches are bolded and underlined, PAM is in green, deletions are highlighted as X in red, and insertions are in red letters. Note: For mouse # 4 in (**b**), the whole deletion is not depicted (to fit the figure)

We have generated six other alleles in the laboratory using six additional sgRNAs by injection of zygotes, and found no other mutations upon screening the top 5 off-target candidates for each sgRNA in all mice with the intended on-target mutation. In contrast to the off-target site with mutations in this study (where all mismatches are outside of the seed region), candidate off-target sites with a PAM for all other sgRNAs –contained ≥3 mismatches with ≥1 mismatches in the seed region. Our results, while rare, provide important and compelling evidence that high-efficiency off-target mutations can occur in mammalian embryos. Our data are consistent with previous observations that this system is more tolerant when mismatches are away from the seed region. Combined, these data underscore the need to identify and screen potential off-target events when engineering genetic models, with an emphasis on sites that have ≤3 mismatches outside of the seed region. Importantly, unlike cell-lines, mice can be backcrossed to remove inter-chromosomal off-target mutations. Finally, this observation needs to be considered as the technology advances for therapeutic purposes.

## Electronic supplementary material

List of candidate off-target sites for the selected sgRNA
