# Supplementary material for: CRISPR/Cas9 can mediate high-efficiency off-target mutations in mice in vivo
Source: Cell Death Dis. 2018 Oct 27;9(11):1099. doi: 10.1038/s41419-018-1146-0 (PMC6204134; doi:10.1038/s41419-018-1146-0)
Supplement: Supplementary file 1 — List of candidate off-target sites for the selected sgRNA [file 41419_2018_1146_MOESM1_ESM.pdf]

guide id	chr	strand	position	sequence	#mismatches	score	ontarget	gene	Comments
25754800	chr12	-1	105932862	GACCCACGGCAGCATTCTCCAGG	0	100	True	NM_148948	
**25754800**	**chr2**	**-1**	**173176074**	**AACACACAGCAGCATTCTCCTGG**	**3**	**2.60631001**	**False**	**None**	**Off-target site with high frequency mutations**
25754800	chr17	-1	83618537	CACCCACTTCAGCATTCTCCTAG	3	1.63277075	False	None	No PAM
25754800	chr7	-1	77922911	CTCCTACAGCAGCATTCTCCAGG	4	1.38618677	False	None	Screened in all 38 animals: No mutations
25754800	chr1	1	91177613	CCCCCACCTCAGCATTCTCCTGG	4	0.86035079	False	None	Screened in 6 mice: No mutations
25754800	chr9	1	4671286	GTCACACTTCAGCATTCTCCTAG	4	0.84696012	False	None	No PAM
25754800	chr4	1	140221339	GTGCCACCTCAGCATTCTCCGGG	4	0.83510268	False	None	Screened in 6 mice: No mutations
25754800	chr16	1	92394088	GGCTCCCAGCAGCATTCTCCAAG	4	0.82579023	False	None	
25754800	chr17	1	91260904	GTCCAGCAGCAGCATTCTCCAAG	4	0.82579023	False	NR_045463	No PAM
25754800	chr6	-1	28804092	GACGGGCAGCAGCATTCTCCGGG	4	0.80123141	False	None	
25754800	chr2	-1	44833566	GTCCAACGTAAGCATTCTCCAGG	4	0.79238308	False	None	
25754800	chr1	-1	41225645	GCCTCCCGGGAGCATTCTCCCAG	4	0.7846019	False	None	
25754800	chr12	-1	15141670	GAGCTACGGAGGCATTCTCCAGG	4	0.70968215	False	None	
25754800	chr17	-1	64466257	GGCACACTGCAGCATTCTCTGAG	4	0.69744718	False	None	
25754800	chr5	-1	142033165	TACCCACGGCAGGATTCTCACAG	3	0.5977	False	None	
25754800	chr3	1	87047946	CACACATGTCAGCATTCTCCCAG	4	0.58761959	False	None	
25754800	chr4	-1	62744391	CAGCCACAGCAGGATTCTCCCAG	4	0.57358054	False	None	
25754800	chr15	1	85378974	TACCCACAGGAGCATTGTCCGGG	4	0.57158781	False	None	
25754800	chr7	-1	127866740	GACCCATGACTGCATTCTCCAAG	3	0.56201349	False	None	
25754800	chr9	1	68179850	GAGCCAGGGCAGCAATCTCCCAG	3	0.53664261	False	None	
25754800	chr3	1	151769258	GAGCCAAGAGAGCATTCTCCCAG	4	0.52531549	False	None	
25754800	chr11	1	7088427	GACTAACTGCAGCATTCTGCCGG	4	0.49875	False	NR_045101	Screened in 6 mice: No mutations
25754800	chr4	-1	95247381	GAGCCAGGGAGGCATTCTCCCAG	4	0.48471291	False	None	
25754800	chr8	-1	130021820	GACCCACAGCAGGATTCTCTGAG	3	0.47984366	False	None	
25754800	chr14	-1	34032787	GTCCCTCAGCAACATTCTCCAGG	4	0.43282194	False	None	
25754800	chr8	-1	47323538	GACTCAAGGGAGCATTCTCAAGG	4	0.42284285	False	None	
25754800	chr3	-1	101949050	GAGGCAGGGCAGCATTTTCCAAG	4	0.40334599	False	NM_009814	
25754800	chr7	-1	91702370	GCCTCAGGGCAGAATTCTCCAGG	4	0.39072347	False	None	
25754800	chr11	1	80762412	TACCCAAGGGAGCATTTTCCAGG	4	0.39039448	False	None	
25754800	chr7	-1	147483848	GGCCAAAGGCAGCATTCTGCAAG	4	0.35320464	False	NM_001128146	
25754800	chr18	-1	76248553	GTCCCAGGGCTCCATTCTCCCAG	4	0.2711862	False	None	
25754800	chr18	1	24619599	GCCCCAAGGAAGCAGTCTCCAAG	4	0.25775914	False	None	
25754800	chr3	1	145296565	CACCCAGGGCACCATTCTCAGAG	4	0.23885286	False	None	
25754800	chr7	1	100050936	GCCCCAGGGCACCATTCTCAGGG	4	0.23436736	False	None	
25754800	chr5	1	93263840	GAACCACGGGATCATTCTTCCAG	4	0.22686881	False	None	
25754800	chr5	1	119213487	GACCCACCGTAGGATTCTCTGAG	4	0.22341544	False	NM_172424	
25754800	chr13	1	41666834	GGGCCACTGCAGCGTTCTCCCAG	4	0.22083592	False	None	
25754800	chr10	-1	61079555	AACACACGGAAGCTTTCTCCAGG	4	0.20981902	False	None	
25754800	chr14	-1	116748243	GGCCCACTGCAGCATTATTCTAG	4	0.19909778	False	None	
25754800	chr19	-1	6464898	CACCCACAACAGCATTCACCAGG	4	0.19660403	False	None	

25754800	chr16	1	31488694	GACCGACAGCAGGATTCTGCTGG	4	0.18964479	False	None	
25754800	chr15	-1	86593286	GACCCAGAGCAACATTGTCCAAG	4	0.18812015	False	None	
25754800	chr17	-1	50368910	ACCCCATGGCAGCATGCTCCCAG	4	0.18600367	False	None	
25754800	chr17	-1	85097435	GACCCTGGGGAGCATTCTGCTGG	4	0.18329272	False	None	
25754800	chr6	1	87285260	GCCCCAGAGCAGCATCCTCCAGG	4	0.18275469	False	NM_054041	
25754800	chr8	1	89060712	GGACCAAGGCAGCATCCTCCAGG	4	0.18019613	False	None	
25754800	chr6	1	15749663	GACCCTCTGCAACATTGTCCTGG	4	0.1694022	False	NM_175088	
25754800	chr15	-1	95975343	GACCCACAGTATCAGTCTCCCAG	4	0.16833766	False	None	
25754800	chr12	-1	9208246	TACCCACACCAGCATGCTCCTGG	4	0.16639567	False	None	
25754800	chr6	-1	6735195	GACTCACGGCAGCAATCACCCAG	3	0.16551111	False	None	
25754800	chr7	-1	35608116	GACCCCAGCCAGCATTCTCAAAG	4	0.16378448	False	None	
25754800	chr12	1	113715104	GGCCCACCACAGCATCCTCCCAG	4	0.16348919	False	None	
25754800	chr16	1	45951733	GACTCACGACAGCATTATCATGG	4	0.15812502	False	None	
25754800	chr18	-1	73467718	GACCCATGGGAGGATTCTCAAAG	4	0.15521236	False	None	
25754800	chr11	1	74847514	GCCACATGGCAGCCTTCTCCCGG	4	0.15297255	False	None	
25754800	chr10	-1	125445586	GCCCCACGGCAGCACACTCCAGG	3	0.14524444	False	NM_177152	
25754800	chr18	-1	25034894	GTCCCAAGGAAGCCTTCTCCAAG	4	0.14088772	False	None	
25754800	chr4	1	102568023	CACCAACGACAGCCTTCTCCTGG	4	0.1391959	False	None	
25754800	chr12	-1	111857056	GTCACACGGCAGCATTCCCAAAG	4	0.13669965	False	None	
25754800	chr5	-1	30452615	GAACCAAGGGAGCTTTCTCCAGG	4	0.13660964	False	None	
25754800	chr10	-1	119855134	GAGGCACGGCAGCATTCCCATAG	4	0.13230132	False	None	
25754800	chr15	1	4031203	GACCCGGGGCTGCATTGTCCTAG	4	0.13051716	False	None	
25754800	chr14	-1	118016990	GTCCCAGGGCAGCAGTCTCATAG	4	0.12766352	False	None	
25754800	chr13	-1	41023739	CACTCACGGCTGCCTTCTCCCAG	4	0.12643817	False	None	
25754800	chr16	1	16869403	GACCCAGGGAAGCATTCTGGGAG	4	0.12633564	False	NM_016982	
25754800	chr16	-1	17980435	GACCCAGGGAAGCATTCTGGGAG	4	0.12633564	False	None	
25754800	chr2	-1	71987007	GCCCCACGCAAGCCTTCTCCAGG	4	0.12603572	False	None	
25754800	chr2	-1	4269649	GACACAAGGCAGCACTCTCGAAG	4	0.12304222	False	None	
25754800	chr19	-1	46922881	GACCCAGGGCTTCATTCTCTCAG	4	0.11890875	False	None	
25754800	chr19	-1	40305280	GACCCACTGCAACATTGTCAGGG	4	0.11873217	False	None	
25754800	chr13	1	21435710	TACCCACGACAGCAGTCTCTCAG	4	0.11639133	False	None	
25754800	chr15	1	51562403	CACCCACGCCAGCACTCTCTGAG	4	0.11639133	False	None	
25754800	chr17	-1	57342282	GACACACAGCACCTTTCTCCAGG	4	0.10659582	False	None	
25754800	chr2	-1	113839793	GACCCTCTGCAGCACTCTCGCAG	4	0.10518302	False	None	
25754800	chr11	-1	95377296	GACCAACAGCATCTTTCTCCCAG	4	0.10488343	False	None	
25754800	chr9	-1	123308868	GACCCACTGTGGCCTTCTCCTGG	4	0.10395688	False	None	
25754800	chr17	1	66206387	GACCCACAGCTCCACTCTCCAGG	4	0.10144126	False	None	
25754800	chr19	-1	47475588	GAACCAGGGCTGCATCCTCCTGG	4	0.09829196	False	None	
25754800	chr2	-1	97217571	GACCCAAGGCCGCATTATCAGAG	4	0.09304851	False	None	
25754800	chr7	1	91021262	GGGCCACGGCAGCCTTGTCCTGG	4	0.08955633	False	None	
25754800	chr1	1	107281746	GACCCACTGAAGCATCTTCCCAG	4	0.08725782	False	None	
25754800	chr14	-1	121518208	AAACCACGGCAGACTTCTCCAGG	4	0.08693069	False	None	
25754800	chr11	1	94050947	GACCCATCCCAGCCTTCTCCCGG	4	0.08619259	False	None	

25754800	chr16	-1	6138303	GACCCATTTCAGCCTTCTCCTGG	4	0.08619259	False	None	
25754800	chr5	1	122426746	GTCCCACAGCAGCAATCGCCAGG	4	0.08467466	False	None	
25754800	chr8	1	28125732	GACACACGACAGCAATCTACAAG	4	0.08166932	False	None	
25754800	chr17	1	67391472	GACCCAGTGCCGCCTTCTCCTGG	4	0.07829278	False	NM_008984	
25754800	chr9	1	104309303	GACACACGGGAGATTTCTCCTGG	4	0.07722282	False	None	
25754800	chr8	-1	41382696	GACCCACATCAGCAGTCTTCCAG	4	0.07624729	False	None	
25754800	chr2	-1	119131413	CACCCACGTCAGCATGCTCTGGG	4	0.07469892	False	None	
25754800	chr12	-1	56436073	GACCCAGGGAAGCATCCTCATAG	4	0.06898327	False	None	
25754800	chr5	-1	131143202	GACCCACCTCAGCATACTCTGAG	4	0.06587373	False	None	
25754800	chr16	-1	23321293	GCCCCACGGCCACTTTCTCCTGG	4	0.06115766	False	NM_145933	
25754800	chr2	1	91145877	GACCGACAGCAGCCGTCTCCAAG	4	0.05806439	False	None	
25754800	chr10	1	21302689	GACCTAGGGCAGCATCCTTCAGG	4	0.05756773	False	None	
25754800	chr2	-1	79915757	GACCCAGGCCTGCATGCTCCAGG	4	0.05699528	False	None	
25754800	chr16	1	86110118	GACCCACTGCTTCCTTCTCCCAG	4	0.05553397	False	None	
25754800	chr12	1	103906238	GTCCAACGGCAGCATGCACCCAG	4	0.05434344	False	None	
25754800	chr11	-1	118540361	GATCCACGACAGCTTTTTCCCAG	4	0.05376313	False	None	
25754800	chr17	-1	14953956	GACCCAAGGCAGTATTCTGAAAG	4	0.05308566	False	None	
25754800	chr9	-1	61441273	GACCCAAGCCAGCACTCTACAGG	4	0.05295583	False	None	
25754800	chr5	-1	114064487	GAACCACCGCAGCATCCGCCTGG	4	0.05263005	False	NM_008153	
25754800	chr1	1	39758265	GACCCACAGCTGCATTCCACAGG	4	0.0506521	False	None	
25754800	chr11	-1	75323837	GCCCCACGGCGGCCTTTTCCAAG	4	0.05040949	False	NM_001029938	
25754800	chr15	-1	7834347	GACCGACGACAGCAATCCCCGGG	4	0.04907169	False	NM_001081402	
25754800	chr4	-1	154613993	GACCCAGGGCAGCCTGCTCCTGG	3	0.04799056	False	None	
25754800	chr7	-1	117939368	GACCCAGGGCATCATTCCCTGGG	4	0.041993	False	NM_001276301	
25754800	chr7	-1	150745743	GGCCCAAGGCAGCTCTCTCCTGG	4	0.04170045	False	None	
25754800	chr11	-1	58936461	CACCCACAGCAGCCTCCTCCAGG	4	0.04057767	False	NM_199152	
25754800	chr5	-1	24259020	GACCCAAGACAGAATCCTCCTAG	4	0.03974265	False	None	
25754800	chr1	1	62406712	GACCCACGGAAGAATCCTCACAG	4	0.03717268	False	None	
25754800	chr14	-1	57960946	GACCGACGTCAGCTCTCTCCGAG	4	0.03547734	False	None	
25754800	chr18	1	74885669	GACCCAGGACAGCATACTACAGG	4	0.03398658	False	None	
25754800	chr2	1	31882398	GACCCACAGCAGCATCCTGTCAG	4	0.03396109	False	NM_172268	
25754800	chr7	1	99534621	GACCCATGGCACCATCCTTCAGG	4	0.02736726	False	None	
25754800	chr4	1	114612743	GACCCAAGGTAGCTTCCTCCAGG	4	0.02306485	False	None	
25754800	chr3	1	116094735	GACCCACAGCAGCAAGCTTCAGG	4	0.02146405	False	None	
25754800	chr7	1	136127324	GACCCTCGGCAGCATCCTAGGGG	4	0.02126424	False	None	
25754800	chr1	1	172631500	GACTCACGGCAACCTCCTCCTGG	4	0.01895337	False	None	
25754800	chr11	1	24567765	GACCCAGGGCATCTGTCTCCCAG	4	0.01889475	False	None	
25754800	chrX	-1	142025099	GACCCAAGGCAGCACTCAGCCAG	4	0.01698747	False	None	
25754800	chr6	1	89269150	GCCCCACGGCAGCAAGCCCCAGG	4	0.01456404	False	NM_008881	
25754800	chr3	1	153445318	GACCCACGGCAGCAGGAACCTGG	4	0.00453913	False	None	
25754800	chr15	-1	72815171	GACCCACGGCAGCTCTTACCTGG	4	0.00399062	False	None	
25754800	chr2	1	118483872	GACCCACGGCAGCCTCTACCAGG	4	0.00256115	False	None	
